# Effect of Comprehensive Educational Program on Preeclamptic Women’s Risk Perception of Cardiovascular Disease, Self-Efficacy, and Adherence to Healthy Lifestyle Behaviors

**DOI:** 10.3390/healthcare12181810

**Published:** 2024-09-10

**Authors:** Nahed Ahmed Hussien, Hend Ali Mohamed Abd El-fatah, Zhenxiang Zhang, Hassanat Ramadan Abdel-Aziz, Ahmad Mahmoud Saleh, Kamala Dhakal, Yongxia Mei, Asmaa Morgan Farahat Khatap

**Affiliations:** 1 Department of Community Nursing, School of Nursing and Health, Zhengzhou University, Zhengzhou 450001, China; nahedahmed@nursing.suez.edu.eg (N.A.H.); kdhakal77us@yahoo.com (K.D.); myx@zzu.edu.cn (Y.M.); 2 Department of Maternity, Obstetrics and Gynecological Nursing, Faculty of Nursing, Suez Canal University, Ismailia 41522, Egypt; hendabdel-fattah@nursing.suez.edu.eg; 3 Department of Nursing Administration and Education, College of Nursing in Al-Kharj, Prince Sattam Bin Abdulaziz University, Al-Kharj 11942, Saudi Arabia; h.abdelrahman@psau.edu.sa (H.R.A.-A.); am.saleh@psau.edu.sa (A.M.S.); 4 Department of women’s Health and Development and Midwifery, Maharajgunj Nursing Campus, Maharajgunj, Kathmandu 44600, Nepal; 5 Department of Maternal-Newborn Health Nursing, College of Nursing in Al-Kharj, Prince Sattam Bin Abdulaziz University, Al-Kharj 11942, Saudi Arabia

**Keywords:** cardiovascular disease, preeclampsia, risk perception, healthy lifestyle, self-efficacy

## Abstract

Purpose: To evaluate the effect of a comprehensive educational program on preeclamptic women’s knowledge, risk perception of cardiovascular disease, self-efficacy, and adherence to healthy lifestyle behaviors. Patients and methods: This study employed a pretest-posttest design. One hundred and two women who previously had preeclampsia were enrolled from July 2022 to December 2022 from outpatient obstetrics, gynecology, and family planning clinics. The primary and secondary outcomes were measured at baseline, after eight weeks, and after three months of the educational intervention. The data were analyzed using SPSS version 23, descriptive and inferential statistics, specifically the Chi-square test, independent *t*-tests, and repeated measures ANOVA. Results: A statistically significant difference was found between the two groups immediately post-intervention and the three-month follow-up, with a significant improvement among the intervention group than control group regarding cardiovascular disease knowledge (*p* < 0.001), risk perception (*p* < 0.001), self-efficacy (*p* < 0.001), and healthy lifestyle behaviors (*p* < 0.001). There was a statistically significant interaction between group and time regarding total cardiovascular disease risk perception (F = 203.67, *p* < 0.001, η2 = 0.673), self-efficacy (F = 70.06, *p* < 0.001, η2 = 0.405), and adherence to healthy lifestyle behaviors (F = 145.08, *p* < 0.001, η2 = 0.597). Conclusion: This study concluded that the comprehensive educational program had a positive effect on improving preeclamptic women’s knowledge and risk perception of CVD, self-efficacy, and adherence to healthy lifestyle behaviors following preeclampsia.

## 1. Introduction

Preeclampsia is a relatively severe type of hypertensive disorder of pregnancy (HDP), defined by high blood pressure (≥140/90 mmHg), that starts to show up after 20 weeks along with proteinuria (≥300 mg/day) or other maternal issues such as uteroplacental dysfunction, neurological, and hematological abnormalities. Preeclampsia complexes 2–8% of pregnancies [1,2,3], leading to maternal and fetal morbidity and mortality and further complications later in life [4,5]. A woman’s risk of cardiovascular disease (CVD) within ten years of delivery is 75% higher if she has had preeclampsia [6,7,8]. During the initial year following childbirth, women who had experienced preeclampsia have a much higher risk of developing CVD, ranging from two to seven times greater than women with normotensive pregnancies. Preeclampsia exhibits a significant correlation with an elevated risk of cardiovascular risk factors (such as dyslipidemia, obesity, and hypertension) [9,10,11] and shares the same pathological changes that affect endothelial function and lead to an increased risk of CVD later in life. According to the Preeclampsia Foundation, women who have preeclampsia are twice as likely to have heart disease and four times as likely to have chronic hypertension [12]. These cardiovascular problems can start during the first 60 days following birth [13] and increase significantly over the first year and ten years after a complicated pregnancy with preeclampsia [14,15]. However, women after preeclampsia are not aware of this association between preeclampsia and CVD, resulting in a lower perception of CVD risk [15,16]. 

Accurate perception of cardiovascular disease risk is vital for preeclamptic women as it is associated with CVD risk-reduction behaviors; it affects how they act in positive ways for their health. If preeclamptic women understand their risk reasonably and scientifically, they could follow the recommended guidelines to lower their CVD risk, including adopting healthy lifestyle behaviors [17,18]. Recent research conducted in 2020, 2022 and 2023 has provided evidence indicating that women with a history of HDP and preeclampsia possess limited awareness and low perception regarding their elevated risk of cardiovascular diseases in the future [19,20,21]. Most women who do not know about the risk of CVD did not receive health-related education from their nurses or obstetricians regarding the risk and prevention of CVD, the importance of adopting healthy lifestyle behaviors, and regular follow-up [20,22]. Early postpartum intervention is crucial to educate preeclamptic women about their heightened cardiovascular disease risk and promote lifestyle changes (such as weight management, smoking cessation, healthy eating, and regular exercise) as effective and economical preventive measures [23].

Adopting healthy lifestyle behaviors can significantly reduce cardiovascular disease risk [24]. The International Society for the Study of Hypertension suggested that it is crucial to provide education to women who have experienced HDP concerning their potential risk of CVD in the future and modifiable risk factors (such as obesity, unhealthy diet, inactivity, and smoking), in addition to regular monitoring of fasting lipids, blood glucose, and blood pressure [25]. After a history of preeclampsia, few women adopted healthy lifestyle behaviors; only 21.7% improved their diet to be healthy, 13.1% practiced exercise, 9.4% stopped smoking, 9% took prescribed medication, and 15.2% had regular health checks with a specialist [26]. Another qualitative study was conducted in 2022 to explore what women with severe preeclampsia thought affected their level of physical activity, as 51% of women were not achieving the recommended weekly amount of moderate-to-vigorous physical activity [27]. Also, the most common areas where preeclamptic women could benefit from health behavior promotion training were fat and sugar consumption, physical activity, dietary fruit, vegetables, water, salt consumption, tobacco and alcoholic consumption. Most women strongly desired an intervention based on a mobile application to include consistently tracking health-related data, being interactive, incorporating behavior modification techniques, giving information, and is tailored to them [28]. 

Many women expressed their desire to live a healthy life after giving birth, but most do not end up doing it [29]. One of the important factors that significantly influences the adoption of healthy lifestyle behaviors is self-efficacy [30]. Albert Bandura defines self-efficacy as “the conviction that one can perform an action deemed necessary to achieve a desired outcome”. The primary sources of self-efficacy are mastery experience, vicarious experience, physiological feedback, and verbal persuasion. Individuals with a high sense of self-efficacy are more expected to follow their doctors’ orders and stick to the medications, nutrition, and exercise regimen they have been given [31]. A limited body of research has investigated the extent of self-efficacy in adhering to healthy behaviors following preeclampsia and interventions to enhance women’s self-efficacy in this context. Previous research has demonstrated that women with preeclampsia have consistently displayed a notable deficiency in their self-efficacy to adhere to recommended dietary and exercise regimens, and this low self-efficacy was found to be a barrier to adopting healthy lifestyle behaviors [27]. This highlights the necessity for healthcare professionals, particularly obstetric nurses and obstetricians, to implement educational programs to boost women’s sense of self-efficacy and encourage healthy practices, ultimately reducing the likelihood of developing heart disease [21]. Therefore, this study aimed to evaluate the effect of a comprehensive educational program on preeclamptic women’s CVD knowledge, risk perception, self-efficacy, and adherence to healthy lifestyle behaviors.

Research Questions:

1. Does the comprehensive educational program improve preeclamptic women’s CVD knowledge and risk perception?
2. Does the comprehensive educational program improve preeclamptic women’s self-efficacy and adherence to healthy lifestyle behaviors?

## 2. Material and Methods

### 2.1. Design

This study employed a pretest-posttest design.

#### 2.1.1. Setting

This study was carried out at Suez Canal University Hospital and Al-Salam MCH Center (outpatient clinics for obstetrics, gynecology, and family planning) in Ismailia City, Egypt.

#### 2.1.2. Participants

The criteria for the included participants were:

1. Women were 18 years or older.
2. Women who had experienced preeclampsia in the last pregnancy within five years post-delivery [28,32].
3. Women who had internet access and could read and write Arabic were also included.

The exclusion criteria were as follows:

1. Women with a history of cardiovascular disease or chronic hypertension.
2. Women with preeclampsia-related fetal deaths were not eligible [33].

#### 2.1.3. Sampling and Sample Size

A purposive sampling technique was used to recruit the study participants. The sample size was calculated using the G power 3.1 version by a priori power analysis with effect size 0.6 for the outcome variable “adherence to healthy lifestyle behaviors”, alpha error prob. = 0.1, and power = 0.89. The total sample size after adding 10% dropout was 102 women who were randomized to two groups in a 1:1 ratio. The intervention group included 51 participants, and the control group included 51 to compare the difference between two unmatched pairs.

#### 2.1.4. Recruitment and Randomization

The women who met the inclusion criteria for participation and were interested in and agreed to participate were provided with details regarding the study aim, the content of the educational program, and its benefits. After that, their written informed consent was taken. The recruitment of participants occurred from July 2022 to December 2022. Using a random Uniform Formula, the participants were randomly allocated to intervention and control groups in a 1:1 ratio. Each group consisted of 51 participants. The data were collected from the participants 3 times, at baseline (T0, face to face), after completing the intervention (T1, online), and the last time after three months of intervention (T2, online). A pilot test was performed on ten percent of the total sample size (with similar socio-demographic characteristics, inclusion and exclusion criteria of the target population) to test the comprehensive educational program content, which was then excluded from the study. 

### 2.2. Intervention

#### 2.2.1. Intervention Group

The researcher (who is an obstetric nurse with a Master’s degree in obstetric nursing and has more than nine years of experience in this field) gave the participants the comprehensive educational program through eight weekly sessions (one per week). The educational sessions were given online using WhatsApp (a social media application for making chat groups and video calls) and Zoom (a meeting program); each session lasted one hour. The researcher created a chat group on WhatsApp containing the researcher and all the intervention group participants. The online session link (Zoom meeting) was sent to the WhatsApp chat group one hour before the session started; each participant could click the link to enter the session. After each session, the educational content was sent to the WhatsApp group to help women remember and follow the educational guidelines. The researcher called each participant the day before the session to confirm her timely attendance. A weekly SMS message was sent to each participant to motivate them to follow healthy lifestyle behaviors, including the benefits of and overcoming barriers to adopting healthy lifestyle behaviors. At the end of the intervention, an electronic educational booklet containing all the educational program content was mailed to each participant.

The educational program was developed based on the Health Belief Model, Bandura’s self-efficacy theory, and different scientific literature such as CVD knowledge and healthy lifestyle content adapted from (Spratling P M et al., 2014; Arnett D K et al., 2019; Bull F C, et al., 2020; Lichtenstein A H et al., 2021; Khosla K et al., 2021; Tsao C W et al., 2022) [33,34,35,36,37,38], relation between preeclampsia and CVD adapted from (Webster R and Heeley E, 2010; Mosca L et al., 2011; Powe C E, Levine R J, and Karumanchi S A, 2011; Say L et al., 2014; Wu P et al., 2017; Arnett D K et al., 2019; Brown H L et al., 2020; Vahedi F A, Gholizadeh L and Heydari M. 2020; Roth H et al., 2021) [15,17,34,39,40,41,42,43,44], and self–efficacy enhancing intervention adapted from (Larson J L et al., 2014; Al Hashmi I H. 2017; Yang X et al., 2022) [45,46,47]. The developed educational program was sent to seven experts with the highest level of education above a master’s degree, with the following qualifications: they have ongoing research projects and are competent in cardiovascular disease and pregnancy hypertensive disorders. The educational program package was modified according to the experts’ suggestions. Then, the educational content was translated into Arabic and transformed into info-graphic forms (static and video info-graphs with the help of a specialist in designing the info-graphs). The educational program covered three main topics: cardiovascular disease knowledge including definitions, risk factors and causes, and prevention methods explaining detailed healthy lifestyle behaviors (session numbers 1, 2, 4, 5); the relation between CVD risk and preeclampsia (session 3); and self–efficacy enhancing intervention for adherence to healthy behaviors through enhancement of mastery experience, verbal persuasion, vicarious experience, and physiological feedback (session numbers 6, 7 and 8).

#### 2.2.2. Control Group

The researcher created a chat group on WhatsApp containing all control group participants. They received no intervention; the participants were required to fill out the data collection questionnaires at baseline, after eight weeks, and after three months for a follow-up. After completing the follow-up test, the electronic educational booklet containing all the educational program content was mailed to each participant.

#### 2.2.3. Quality Control

The quality of the study was controlled through the following:

1. Measurement of participants’ adherence to the intervention: The number of attended sessions and completed tasks in the worksheet were used to determine adherence to the intervention. The researcher reminded the participants of the intervention schedule the day before each session. If a participant missed a session, the researcher contacted her via telephone to determine the cause and provide a concise summary of the session. Alternatively, the session was recorded with the “did not attend”. Non-adherence to the intervention was predetermined by a *Priori* as attending less than 80% of the sessions [48].

2. The statistician randomly assigned participants using a Random Uniform Formula, and the tools used throughout the study were reliable and valid.

3. Information exchange between the intervention and control groups was prevented by ensuring that each group used a different chat group, and the intervention group participants were instructed not to share the educational material with others.

#### 2.2.4. Outcome Measures

Participants’ demographic, clinical, and historical data (age, marital status, educational level, residence, income, employment, BMI, smoking and alcohol intake, family history of chronic disease, and preeclampsia history) were collected using initial interviews and medical records.

1. Primary outcomes:

-Risk perception of cardiovascular disease was assessed using the Perception of Risk of Heart Disease Scale (PRHDS). This self-reported scale was utilized with the author’s official consent to be employed in Arabic and English [49]. The 20 elements were broken down into three subscales: dread risk, risk, and unknown risk. The Likert scale allows respondents to rate each statement from one (indicating strongly disagree) to four (indicating strongly agree). The results of each subscale are aggregated to calculate the overall score, and higher scores on the PRHDS subscale reflect a greater perception of the risk of developing heart disease. The PRHDS has demonstrated strong construct validity in the past, with an overall scale alpha of 0.80 [49] and a reliability coefficient of 0.86 on its scale in our previous study [50]. 

-The adherence to healthy lifestyle behaviors was assessed by Health-Promoting Lifestyle Profile II (HPLP-II); it was made by Walker et al. [51] and translated into Arabic by Eshah et al. [52]. The authors permitted the researcher to use the Arabic and English versions [52]. The current study described a healthy lifestyle as the pattern of daily decisions that will positively affect nutrition, spiritual growth, interpersonal relationships, stress management, health responsibility, and physical activity [35]. The HPLP-II comprised 52 behavior statements that used a 4-point Likert scale response format (never = 1, sometimes = 2, often = 3, and routinely = 4). Six subscales measure key aspects of a healthy lifestyle, including spiritual growth (9 items), stress management (8 items), health responsibility (9 items), physical exercise (8 items), nutrition (9 items), and relationships (9 items). For scoring, means can be calculated independently for each subscale or the whole instrument to measure how HPLP-II is used. A higher total or subscale mean would show that HPLP-II is more adopted [53] (a total score ≥ 104 is considered a high adoption of healthy lifestyle behaviors, and a total score ˂ 104 is considered a low level of adoption of healthy lifestyle behaviors). Earlier studies in the Jordanian population used a similar scale, reporting high internal consistency reliability (a = 0.89, 0.91, 0.93, and 0.89, respectively) [52,53,54,55]. Cronbach’s alpha was recalculated in this study and found to be 0.85.

2. Secondary outcomes:

-Cardiovascular disease knowledge was assessed by the Adapted Coronary Heart Disease knowledge tool for PE women [50]. The questionnaire has 26 true and false questions about CVD risk factors and preventative strategies. Accurate answers were worth 1 point, whereas false or “I do not know” answers were worth 0 points. Scores varied from 0 to 26, with greater scores indicating greater knowledge level [33,56]. In our prior research, the questionnaire’s content and face validity were high, and Cronbach’s alpha was 0.90 [50].

-Self-efficacy for adherence to healthy lifestyle behaviors was assessed by health-specific self-efficacy scales, which were developed by [57,58], and the authors said researchers could use them without permission; it is available for all researchers. Three scales were used to measure self-efficacy for healthy behavior adherence via a 4-point Likert scale (from 1 = very uncertain to 4 = very certain) and a composite score ranging from 13 to 52 based on responses to all 13 items. The three scales were a five-item Nutrition self-efficacy scale, a five-item Physical Exercise self-efficacy scale, and a three-item Alcohol Resistance self-efficacy scale. A high score on the scale signified a substantial degree of self-efficacy. Cronbach’s alpha for health-specific self-efficacy scales was measured in the current study; it was 0.92.

### 2.3. Data Analysis

The data were analyzed using SPSS version 23. Descriptive statistics (e.g., percentage, frequency, mean, and standard deviation) were used to report the socio-demographic and historical characteristics of the participants, and inferential statistics (including independent *t*-tests, repeated measures ANOVA (F-Test), and Chi-square test) were applied. The *p*-value was significant <0.05.

### 2.4. Ethical Considerations

The study was carried out in compliance with the ethical guidelines outlined by the Declaration of Helsinki. The ethical committees at Suez Canal University in Egypt (No. 144/2022) and Zhengzhou University in China (IRB 2021-134) have approved all human experiments in this study. Participants who volunteered to participate in the study provided written informed consent. Every participant had the right to discontinue involvement in the research at any given point.

## 3. Results

**Figure 1:**
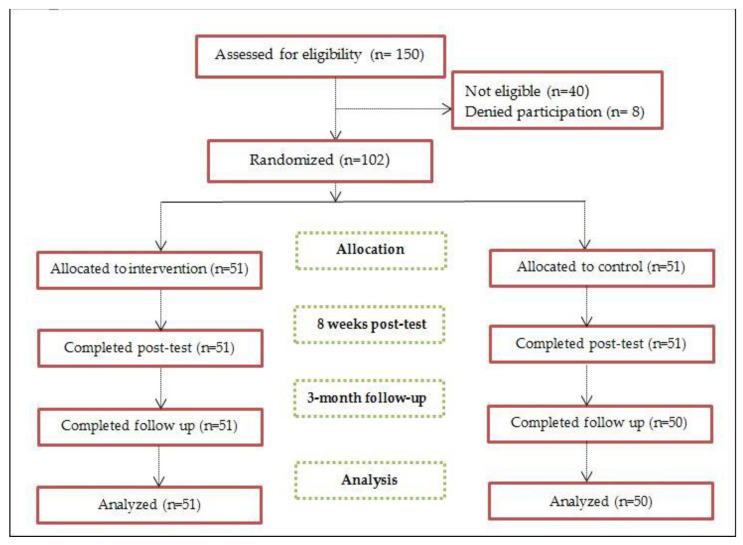
The flow diagram of the study.

Among 150 participants evaluated for eligibility, 40 participants were not eligible (did not meet the inclusion criteria), and 8 participants were denied participation in the study. Therefore, the total number of the included participants in this study was 102, and they consented to participate in this study and were randomly assigned to control and intervention groups. The study flow diagram is illustrated in Figure 1. Among 51 participants in the intervention group, 96% attended all sessions and completed the assignments. Two participants attended seven sessions and accomplished the assignments. Intervention adherence was above a priori adherence criterion of 80% of sessions.

The demographic characteristics of the participants in both groups are presented in Table 1. The mean age of participants in the intervention group was 31.82 ± 6.13, and the control group was 31.29 ± 7.56. There was no statistically significant difference between the intervention and control groups regarding education level, employment, residence, BMI, and smoking, with *p* > 0.05. However, a statistically significant difference was found between both groups concerning marital status (*p* = 0.004) and income (*p* < 0.001).

Table 2 displays the study participants’ family and preeclampsia history. Regarding family history, there was a statistically significant difference (*p* < 0.001) between the control and intervention groups. At the same time, there was no statistically significant difference between the two groups concerning pregnancy complications, awareness of the link between CVD and preeclampsia, source of knowledge, and the doctor’s discussion of CVD risk with women with *p* > 0.05.

Table 3 reveals no statistically significant difference between the two groups in terms of total cardiovascular disease risk perception at baseline. Cardiovascular disease risk perception improved significantly in the intervention group following the intervention and follow-up compared to the control group with a *p* < 0.001, and the intervention group’s mean of cardiovascular disease risk perception scores increased from (35.30 ± 6.83) at baseline to (54.53 ± 5.62) at 3 months post-intervention. A statistically significant interaction was found between group and time related to total cardiovascular disease risk perception (F = 203.67, *p* < 0.001, η2 = 0.673).

Regarding CVD knowledge, Table 3 presents that the level of cardiovascular disease knowledge improved significantly following the intervention among the intervention group, with no improvement among the control group (*p* < 0.001). A statistically significant interaction was found between group and time related to total adherence to healthy lifestyle behaviors (F = 120.47, *p* < 0.001, η2 = 0.517).

Regarding total self-efficacy and its dimensions, Table 3 shows no statistically significant differences between the two groups at baseline. Comparing the intervention group to the control group, the total self-efficacy for adherence to healthy lifestyle behaviors improved significantly following the intervention among the intervention group with (*p* < 0.001); the mean scores of total self-efficacy increased from (26.72 ± 5.01) at baseline to (38.43 ± 4.80) at 3 months post-intervention. There was a statistically significant interaction between group and time regarding total self-efficacy (F = 70.06, *p* < 0.001, η2 = 0.405).

Table 4 represents a statistically significant difference between the two groups in terms of total adherence to healthy lifestyle behaviors and its subscales post-intervention and in the 3-month follow-up compared with no difference at baseline. A significant improvement was found among intervention group participants following the intervention and follow-up compared to the control group with *p* < 0.001. The intervention group’s mean scores of total adherence to healthy lifestyle behaviors increased from (101.7 ± 11.76) at baseline to (142.2 ± 16.92) at 3 months post-intervention. A statistically significant interaction between group and time was related to total adherence to healthy lifestyle behaviors (F = 145.08, *p* < 0.001, η2 = 0.597).

## 4. Discussion

The present study was conducted to evaluate the effect of the comprehensive educational program on women’s knowledge, risk perception of CVD, self-efficacy, and adherence to healthy lifestyle behaviors after preeclampsia. Our comprehensive educational program included eight educational sessions explaining cardiovascular disease signs and symptoms, risk factors and causes, and healthy lifestyle behaviors as an effective strategy to reduce the risk of CVD. In addition, our comprehensive educational program addressed the relationship between preeclampsia and the risk of CVD in the future. Finally, we provided a self-efficacy-enhancing intervention for adherence to healthy behaviors by strengthening the four sources of self-efficacy including mastery experience, vicarious experience, verbal persuasion, and physiological feedback. We provided our intervention online, contributing to a high adherence rate to our comprehensive educational program. Preeclampsia survivors preferred web-based interventions; these interventions effectively engaged women at home, were more flexible, and better fit the mothers’ unpredictable schedules, especially young and employed mothers, who showed high attendance rates to the intervention sessions [32,59]. 

Concerning CVD knowledge, the current research elucidated that following the execution of the comprehensive educational program, there was a noteworthy statistically significant difference between the control and intervention groups immediately post-intervention and three months later compared with no difference in the baseline regarding knowledge of CVD. The intervention group’s CVD knowledge was significantly improved. These results are in accordance with Rich-Edwards et al. [32], who found that after nine months of online education, there was a significant improvement among intervention group participants regarding knowledge of CVD risk factors [32]. Another research conducted by Spratling et al. [33] supported our results, where they studied the impact of telephone-based education on risk perception of CVD among preeclamptic women. They found that after the application of telephone educational intervention, women’s knowledge of risk factors was improved compared with baseline data [33]. Moreover, Parfenova et al. [60] performed a Randomized Trial using an educational pamphlet to improve women’s knowledge of health risks after HDP; after one month of providing pamphlet education, the intervention group demonstrated a higher level of knowledge compared to the control group [60]. Furthermore, it was noted that a small number of participants in both groups of the current study received education from their obstetricians. The findings align with the study conducted by Rossiter et al. [61], which revealed that a small number of women mentioned that their general practitioner discussed the risk of CVD with them [61]. Another study agreed with our results: Of the 438 women studied, 62.6% did not know the cardiovascular risks associated with a history of preeclampsia or gestational hypertension. Furthermore, only 53.4% of those who knew the risks had been educated by a doctor or midwife at their birthing hospital [19]. This underscores the significance of the need for obstetricians and midwives to provide health education for women with previous preeclampsia to improve their understanding of their CVD risk.

The improvement of participants’ knowledge in the current study might be due to the educational materials used in this study that were provided to the participants after each session. Also, the electronic educational booklet containing all the sessions’ educational content was mailed to each participant at the end of eight sessions. It helped them retain CVD knowledge and follow the recommended guidelines about healthy lifestyle behaviors. This interpretation is supported by Roth et al. [44], who stated that women who experienced HDP prefer to receive health education during the early postpartum period about their future long-term health risks from their healthcare provider and receive web-based or printed information to take home to help them in knowledge retention and adhere to a follow-up schedule [44].

Regarding the perception of CVD risk, the present study findings revealed a statistically significant difference between the control and intervention groups immediately post-intervention and three months later, compared with no difference in the baseline data. The comprehensive educational program effectively improved the CVD risk perception of participants in the intervention group. These findings are similar to previous research conducted an educational intervention to improve preeclamptic women’s perception of CVD and concluded that the women’s risk perception was significantly improved after the intervention compared to pre-intervention [33]. Along the same line, a postnatal intervention in 2022 aimed to improve women’s health behavior after a complicated pregnancy with HPD found that the participants in intervention groups demonstrated higher improvement in their perception of cardiovascular disease risk after intervention [61].

There is a statistically significant association between knowledge of CVD and perception of CVD risk [50], and patients who lack sufficient knowledge about CVD display a lower risk perception of CVD [62]. This might clarify why the intervention group in the present study had a better perception of CVD. Those who attended the educational intervention gained significant knowledge about CVD, accounting for their elevated perception of CVD risk.

In terms of self-efficacy for adherence to healthy lifestyle behaviors, the present study results showed that the participants in the intervention group gained a higher sense of self-efficacy for adherence to physical exercise and healthy nutrition after the application of the comprehensive educational program when compared with no improvement among participants in the control group with a statistically significant difference between the two groups. This result is in accordance with a previous randomized controlled trial conducted in 2019, which revealed that intervention participants exhibited significantly increased self-efficacy for physical activity and healthy eating compared to the control group [32]. Our results could be illustrated by the fact that self-efficacy is driven by four primary sources: vicarious experience, mastery experience, social persuasion, and physiological status [31], and our intervention focused on enhancing these four sources through different strategies such as goal setting to improve mastery experience, role modeling of different aspects of healthy lifestyle behaviors through video clips, motivational messages, positive feedback on each participants’ goal achievements, and interpretation of physical and emotional body reactions. These strategies helped women in the intervention group gain a sense of self-efficacy for adherence to healthy behaviors. It is important to mention that there have been limited studies that specifically examine the evaluation and enhancement of self-efficacy in preeclamptic women for adhering to healthy behaviors. Therefore, it is recommended to conduct additional research to evaluate the extent of self-efficacy among women who have experienced preeclampsia and to develop strategies to enhance their self-efficacy.

Concerning adherence to healthy lifestyle behaviors, a statistically significant difference was found between the control and intervention groups immediately post-intervention and three months later, compared with no difference in the baseline data. Also, a significant improvement in the adherence to healthy lifestyle behaviors (nutrition, physical activity, health responsibility, spiritual growth, interpersonal relation, and stress management) was found among participants in the intervention group after receiving the comprehensive educational program compared with no improvement among participants in the control group. These findings are consistent with Janmohamed et al. [26], who set up an interdisciplinary clinic to educate postpartum mothers about healthy lifestyle changes to reduce their risk of cardiovascular disease. They found that after six months of counseling and health education about healthy lifestyle behaviors for preeclamptic women, adoption of physical activity significantly improved as 76% of women engaged in physical activity compared with 14% before counseling [26]. Moreover, Rossiter et al. [61] support our findings that after the implementation of counseling from lifestyle coaching and education, participants in the intervention group reported more positive changes in adopting healthy eating and exercise habits [61]. Furthermore, Berks et al. [63] agree with our results when studying the CVD risk after preeclampsia and the effectiveness of lifestyle interventions. They found that participants exhibited lifestyle changes, especially diet, exercise, and smoking cessation following preeclampsia, and decreased cardiovascular risk, either ischemic or stroke [63]. Also, Riemer et al. [64], who implemented an educational intervention to decrease CVD risk, found a high adherence rate among studied participants to nutritional counseling (73%) and the intensive cardiovascular exercise program (79%) [64].

There are two possible explanations for why women in this study were able to increase their compliance with healthy lifestyle behaviors. The first one is that women with a preeclampsia history were concerned with knowing about healthy lifestyle behaviors and were motivated to adopt healthy lifestyle behaviors by the knowledge of the link between CVD and a history of preeclampsia [65]. The second reason is that our educational intervention content meets preeclamptic women’s educational needs regarding healthy habits, especially nutrition, physical exercise, and emotional well-being, as reported by Kókai et al. [66]. Our educational intervention provided detailed information about healthy nutrition, different types of physical activities, in addition to various strategies of stress management, such as relaxation techniques [66]. 

Moreover, accurate risk perception of cardiovascular disease is critical for adopting healthy lifestyle behaviors and motivates the application of healthy lifestyle modifications [67]. In 2023, a study was conducted to see how preeclamptic women who knew about their long-term cardiovascular risks changed their health-seeking behaviors after giving birth. The results showed that women who knew about their CVD risk were more likely to have regular checkups for their blood pressure, cholesterol, and blood glucose [19]. Also, our findings agree with those of Bokslag et al., who investigated the influence of framing and perceived the probability of changing behavior to lower cardiovascular risk after preeclampsia. Nurses specializing in obstetrics and gynecology were recruited for this study because they work with pregnant women and are familiar with the symptoms of preeclampsia. They discovered that one’s level of concern about developing CVD had a significant impact on one’s propensity to make lifestyle changes. According to the Health Belief Model, people are more likely to change their behavior if they think the risk is high [68]. This also could clarify the current study’s improvement among participants who exhibited increased risk perception of CVD when adopting healthy lifestyle behaviors. Therefore, it is essential for healthcare providers, especially nurses and obstetricians, to work to raise women’s awareness of their future risk of CVD and inspire them to employ healthy lifestyle behaviors [67].

It was observed that the adherence rate to our comprehensive educational intervention was high among intervention group participants as it was conducted online. This result aligns with a previous study that reported a high adherence percentage to its online intervention [32]. This may be because women with preeclampsia were curious about a web-based program designed to teach them how to reduce their risk of cardiovascular disease through lifestyle changes. Web-based intervention helps overcome common barriers that prevent women from adhering to face-to-face intervention, such as lack of time and family responsibilities [22,65]. Web-based therapies offer increased flexibility, making them more compatible with the rigorous and unpredictable schedules commonly faced by young and employed mothers. Furthermore, the existing literature has demonstrated that internet-based therapies can improve compliance with interventions and facilitate the adoption of health-promoting behaviors in women who have experienced preeclampsia in the past [28,59].

### 4.1. Study Strengths

This study represents the inaugural interventional study in Egypt that offers a comprehensive educational program to enhance women’s perception of CVD risk after preeclampsia and strengthen their self-efficacy in adhering to healthy -lifestyle behaviors. It also provides nursing researchers worldwide with new findings and baseline data. The comprehensive educational program added a novel component to its content that is lacking in the existing literature by integrating self-efficacy-enhancing intervention and motivation counseling into the comprehensive educational intervention aimed to enhance engagement and long-term adherence to healthy lifestyle behaviors after a complicated pregnancy with preeclampsia. This study is considered a valuable contribution to the field of studying and understanding self-efficacy for adherence to healthy behaviors among women who had preeclampsia and filled significant research gaps globally, as the studies on this topic are few. This study has identified a significant insufficiency in pre-discharge health education provided by nurses and obstetricians about cardiovascular disease risk for women who experienced preeclampsia in Egypt. Therefore, the findings of this study can draw the attention of our nurses and obstetricians towards integrating sufficient content in the pre-discharge education on long-term complications of preeclampsia, especially CVD risk. Also, this study was conducted online, which helped women save time on transportation and acted as a solution for other barriers preventing them from attending a face-to-face intervention. Therefore, the adherence rate to the intervention was high.

### 4.2. Study Limitations

The first limitation was that the participants were from one city; therefore, these findings may not be generalized to other geographical areas in Egypt. The second limitation was that improvement in the adherence to healthy lifestyle behaviors is self-reported. The third limitation was the restricted availability of prior research on self-efficacy for adherence to healthy lifestyle behaviors among women who have experienced preeclampsia, which hindered our ability to make direct comparisons with our study’s outcomes. The fourth limitation was the inability to control the confounding variables, such as additional health-related behaviors or activities on social media.

## 5. Conclusions

Based on the findings of this study, it was concluded that the comprehensive educational program had a positive effect on improving preeclamptic women’s knowledge and risk perception of CVD, self-efficacy, and adherence to healthy lifestyle behaviors following preeclampsia. It is recommended that obstetricians and nurses incorporate a comprehensive educational program in postpartum education of women with a history of preeclampsia to improve their risk perception of CVD, boost their self-efficacy, and encourage them to adopt healthy lifestyle behaviors. As a result of the positive effect of the comprehensive educational intervention, additional studies should be conducted on a larger sample from different geographical areas and over a long follow-up period to generalize the results.

## Figures and Tables

**Table 1:** Distribution of intervention and control groups regarding their socio-demographic characteristics.

Socio-Demographic Characteristics	Intervention Group	Control Group	X^2^ (*p*-Value)
	N = 51	N = 50	
	Number (%)	Number (%)	
**Age (years)**			
18-<22	- (-)	2 (4)	
22-<26	12 (23.5)	9 (18)	
26-<30	7 (13.7)	8 (16)	2.01 ^$^ (0.089)
30-<34	10 (19.6)	13 (26)	
≥34	22 (43.1)	18 (36)	
Mean ± SD	31.82 ± 6.13	31.29 ± 7.56	0.388 (0.699) ^#^
**Education**			
Basic education	11 (21.6)	10 (20)	
Secondary education	23 (45.1)	18 (36)	1.55 ^$^ (0.459)
University education	17 (33.3)	22 (44)	
**Residence**			
Rural	11 (21.6)	8 (16)	0.243 ^$^ (0.445)
Urban	40 (78.4)	42(84)	
**Body Mass Index (BMI)**			
Normal: 18.5–24.9	10 (19.6)	12 (24)	
Overweight: 25–29.9	27 (52.9)	25 (50)	0.259 ^$^ (0.879)
Obese: more than 30	14 (27.5)	13 (26)	
**Income**			
Sufficient ^a^	18 (35.3)	37 (74)	14.24 ^$^ (<0.001)
Insufficient	33 (64.7)	13 (26)	
**Employment**			
Yes	12 (23.5)		1.70 ^$^ (0.192)
No	39 (76.5)	17 (34)	
**Marital status**		33 (66)	
Married	41 (80.4)	49 (98.0)	8.45 ^$^ (0.004)
Divorced	10 (19.6)	1 (2.0)	
**Smoking**			
Yes	-	-	2.00 ^$^ (0.368)
No	51 (100)	50 (100)	
**Alcohol intake**			
Yes	-	-	2.00 ^$^ (0.368)
No	51 (100)	50 (100)	

# is independent *t*-test, $ is Chi-square test, *p*-value is significant <0.05, a is sufficient monthly income ≥ 4000 EGP.

**Table 2:** Distribution of intervention and control groups according to their historical characteristics.

History	Intervention Group	Control Group	X^2^ (*p*-Value)
	N = 51	N = 50	
	Number (%)	Number (%)	
**Family history**			
Hypertension	8 (15.7)	5 (10)	
Diabetes	19 (37.3)	9 (18)	
Heart failure	11 (21.6)	1 (2)	
Stroke	-	-	32.40 ^$^ (<0.001)
Hyperlipidemia	-	-	
Renal failure	1 (2)	-	
None	10 (19.6)	26 (52)	
**Obstetric history**			
**Pregnancy complication**			
Preeclampsia	46 (90)	49 (98)	2.83 ^$^ (0.092)
Eclampsia	5 (9.8)	1(2)	
**Preeclampsia in which pregnancy**			
First	16 (31.4)	18 (36)	
Second	11 (21.6)	14 (28)	
Third	15 (29.4)	7 (14)	6.84 ^$^ (0.446)
Fourth	6 (11.8)	6 (12)	
Fifth	3 (5.9)	2 (4)	
Sixth	-	1 (2)	
**Did the doctor discuss the risk of CVD with you?**			
Yes	42 (82.4)	46 (90)	
No	9 (17.6)	3 (5.9)	5.18 ^$^ (0.075)
I do not remember	-	1 (2)	
**Are you aware of the link between CVD and preeclampsia?**			
Yes	6 (11.8)	4 (7.8)	2.98 ^$^ (0.084)
No	45 (88.2)	46 (92)	
**If yes, please specify the origin of your knowledge.**			
Obstetrician	5 (9.8)	3 (5.8)	
Nurse	-	-	5.69 ^$^ (0.127)
Internet	1 (1.9)	1 (1.9)	

$ is Chi-square test, *p*-value is significant <0.05.

**Table 3:** Comparison between intervention and control groups according to their CVD knowledge, risk perception, and self-efficacy for adherence to healthy lifestyle behaviors.

Items	Intervention Group N = 51 Mean ± SD	Control Group N = 50 Mean ± SD	Sig. ^b^	F	df	*p*-Value	η2
**Total CVD knowledge**							
Pre-intervention	10.52 ± 5.18	11.63 ± 6.49	0.664	120.47	1.06	<0.001	0.517
Post-intervention	22.97 ± 2.24	11.27 ± 6.58	<0.001				
Follow-up intervention	23.20 ± 1.92	11.02 ± 6.35	<0.001				
**Total CVD risk perception**							
Pre-intervention	35.30 ± 6.83	36.90 ± 5.85	0.947	203.67	1.030	<0.001	0.673
Post-intervention	53.68 ± 5.32	36.90 ± 5.85	<0.001				
Follow-up intervention	54.53 ± 5.62	36.90 ± 5.89	<0.001				
**Total self-efficacy**							
Pre-intervention	26.72 ± 5.01	27.29 ± 6.24	0.411	70.06	1.64	<0.001	0.405
Post-intervention	37.78 ± 4.79	26.86 ± 4.98	<0.001				
Follow-up intervention	38.43 ± 4.80	26.73 ± 5.016	<0.001				
**Self-efficacy subscales**							
**Self-efficacy for Healthy Nutrition subscale**							
Pre-intervention	8.10 ± 3.43	8.53 ± 4.23	0.091	62.11	1.65	<0.001	0.383
Post-intervention	13.39 ± 2.31	7.80 ± 2.89	<0.001				
Follow-up intervention	13.61 ± 2.341	7.71 ± 2.91	<0.001				
**Self-efficacy for Physical Exercise subscale**							
Pre-intervention	7.09 ± 2.70	7.06 ± 2.87	0.491	58.60	1.59	<0.001	0.370
Post-intervention	12.39 ± 2.59	7.18 ± 2.69	<0.001				
Follow-up intervention	12.82 ± 2.61	7.10 ± 2.70	<0.001				
**Self-efficacy for Alcohol Resistance subscale**							
Pre-intervention	11.92 ± 0.39	11.71 ± 0.90	0.156	0.329	1.00	0.763	0.003
Post-intervention	12.00 ± 0.00	11.88 ± 0.48	0.080				
Follow-up intervention	12.00 ± 0.00	11.92 ± 0.39	0.156				

F-test is repeated measures ANOVA, *p*-value is significant <0.05, η2 is Partial Eta Squared; b—Adjustment for multiple comparisons: Least Significant Difference (equivalent to no adjustments).

**Table 4:** Comparison between the intervention and control groups according to their healthy lifestyle behaviors.

Total Health-Promoting Lifestyle Profile II and Its Subscales	Intervention Group Mean ± SD	Control Group Mean ± SD	Sig. ^b^	F	df	*p*-Value	η2
**Health responsibility subscale**							
Pre-intervention	15.94 ± 2.32	16.08 ± 4.09	0.835	54.90	1.081	<0.001	0.354
Post-intervention	21.41 ± 3.26	16.19 ± 3.66	<0.001				
Follow-up intervention	21.61 ± 3.44	16.24 ± 3.69	<0.001				
**Physical activity subscale**							
Pre-intervention	10.16 ± 2.05	10.76 ± 3.13	0.725	178.49	1.074	<0.001	0.643
Post-intervention	18.98 ± 3.65	10.98 ± 3.21	<0.001				
Follow-up intervention	19.28 ± 3.71	10.98 ± 3.21	<0.001				
**Nutrition subscale**							
Pre-intervention	18.35 ± 2.91	18.19 ± 3.10	0.156	45.51	1.045	<0.001	0.315
Post-intervention	23.66 ± 3.73	16.92 ± 2.72	<0.001				
Follow-up intervention	24.34 ± 3.66	16.92 ± 2.72	<0.001				
**Interpersonal relation subscale**							
Pre-intervention	21.61 ± 2.88	20.76 ± 2.95	0.148	19.15	1.017	<0.001	0.161
Post-intervention	27.49 ± 3.08	23.73 ± 3.34	<0.001				
Follow-up intervention	27.69 ± 2.99	23.72 ± 3.34	<0.001				
**Spiritual growth subscale**							
Pre-intervention	22.31 ± 3.19	22.96 ± 3.52	0.312	79.22	1.027	<0.001	0.442
Post-intervention	26.88 ± 2.65	22.63 ± 2.88	<0.001				
Follow-up intervention	26.96 ± 2.88	22.63 ± 2.88	<0.001				
**Stress management subscale**							
Pre-intervention	15.53 ± 3.63	15.88 ± 3.64	0.602	105.03	1.026	<0.001	0.512
Post-intervention	21.84 ± 2.91	16.08 ± 3.67	<0.001				
Follow-up intervention	22.14 ± 2.82	16.08 ± 3.67	<0.001				
**Total Health-Promoting Lifestyle Profile II**							
Pre-intervention	101.7 ± 11.76	104.7 ± 14.16	0.206	145.08	1.017	<0.001	0.597
Post-intervention	140.3 ± 16.94	106.5 ± 12.73	<0.001				
Follow-up intervention	142.2 ± 16.92	106.6 ± 12.72	<0.001				

F-test is repeated measures ANOVA, *p*-value is significant <0.05, η2 is Partial Eta Squared; b—Adjustment for multiple comparisons: Least Significant Difference (equivalent to no adjustments).

## Data Availability

The data used and analyzed during this study are available from the corresponding author upon reasonable request.
